# Evaluating AI Ambient Voice Technology as a Documentation Assistant in Psychiatry – a Proof of Concept Study

**DOI:** 10.1192/bjo.2025.10089

**Published:** 2025-06-20

**Authors:** Noah Stanton, Aadam Aziz, Solomon Wong, Salim Jakhra, Sirous Golchinheydari

**Affiliations:** Central & North West London NHS Foundation Trust, London, United Kingdom

## Abstract

**Aims:** Artificial Intelligence Ambient Voice Technology (AI AVT) which uses a large language model to summarise clinical dialogue into electronic notes and GP letters has emerged. Although effective in general practice and medical settings, its potential in psychiatry is unknown. In this proof of concept study, we sought to apply AI AVT into clinical practice for a limited duration.

The specific aims were to:

Assess the functionality and suitability of AI AVT in a child and adolescent mental health service (CAMHS) outpatient clinic for the selected use cases.

Identify whether AI AVT reduces documentation burden during and after clinical consultations, and improves clinician work satisfaction.

Identify whether AI AVT is acceptable to patients.

Identify potential challenges and issues from a clinician, organisational and patient perspective and to make recommendations for refinements.

**Methods:** We conducted a mixed-methods pre-post (manual versus AVT-assisted documentation) service development pilot with 10 clinician participants in a CAMHS outpatient clinic. Use cases were attention deficit hyperactivity disorder medication reviews, general medical reviews and developmental history assessments. The primary outcome was time taken to complete administrative tasks per patient. Secondary outcomes included qualitative clinician experience and patient/carer perception and acceptability of AVT. Measures including questionnaires, time sheets and focus groups were conducted at baseline and intervention. Data analysis included descriptive statistics and mixed linear regression. Focus groups were audio-recorded before being transcribed and thematically analysed.

**Results:** AVT was used in 351 clinical encounters. Administration time for 251 encounters was recorded (AVT n=171). The median time per encounter reduced from 27 minutes (manual) to 10 minutes (AVT) (p<0.001). On average, AVT-assisted documentation took 45% of the time of manual documentation (p<0.001). Clinician-rated accuracy, quality and efficiency of AVT-assisted documentation was statistically significant in its favour. Patient acceptance was high: only 3 preferred for AVT not to be used (0.85%). 97% felt clinicians were not distracted by taking notes. Thematic analysis from focus groups identified positive effects from AVT (improved productivity and mental wellbeing) balanced by barriers (technological limitations).

**Conclusion:** Although subject to the limitations of a small pilot study, we demonstrated that AVT can be implemented successfully, resulting in significantly reduced documentation burden. To evaluate its scalability and potential to further streamline processes, we are currently in phase 2 which involves expanding the clinical roles of our participants and the use cases across Central & North West London NHS Foundation Trust (5 boroughs).

